# Maternal influenza immunization in Malawi: Piloting a maternal influenza immunization program costing tool by examining a prospective program

**DOI:** 10.1371/journal.pone.0190006

**Published:** 2017-12-27

**Authors:** Clint Pecenka, Spy Munthali, Paul Chunga, Ann Levin, Win Morgan, Philipp Lambach, Niranjan Bhat, Kathleen M. Neuzil, Justin R. Ortiz, Raymond Hutubessy

**Affiliations:** 1 Center for Vaccine Innovation and Access, PATH, Seattle, Washington, United States of America; 2 Chancellor’s College, Zomba, Malawi; 3 Ministry of Health, Lilongwe, Malawi; 4 Levin and Morgan, Washington, DC, United States of America; 5 Initiative for Vaccine Research, World Health Organization, Geneva, Switzerland; 6 Center for Vaccine Development, University of Maryland School of Medicine, Baltimore, Maryland, United States of America; Monash University, Australia, AUSTRALIA

## Abstract

**Background:**

This costing study in Malawi is a first evaluation of a Maternal Influenza Immunization Program Costing Tool (Costing Tool) for maternal immunization. The tool was designed to help low- and middle-income countries plan for maternal influenza immunization programs that differ from infant vaccination programs because of differences in the target population and potential differences in delivery strategy or venue.

**Methods:**

This analysis examines the incremental costs of a prospective seasonal maternal influenza immunization program that is added to a successful routine childhood immunization and antenatal care program. The Costing Tool estimates financial and economic costs for different vaccine delivery scenarios for each of the major components of the expanded immunization program.

**Results:**

In our base scenario, which specifies a donated single dose pre-filled vaccine formulation, the total financial cost of a program that would reach 2.3 million women is approximately $1.2 million over five years. The economic cost of the program, including the donated vaccine, is $10.4 million over the same period. The financial and economic costs per immunized pregnancy are $0.52 and $4.58, respectively. Other scenarios examine lower vaccine uptake, reaching 1.2 million women, and a vaccine purchased at $2.80 per dose with an alternative presentation.

**Conclusion:**

This study estimates the financial and economic costs associated with a prospective maternal influenza immunization program in a low-income country. In some scenarios, the incremental delivery cost of a maternal influenza immunization program may be as low as some estimates of childhood vaccination programs, assuming the routine childhood immunization and antenatal care systems are capable of serving as the platform for an additional vaccination program. However, purchasing influenza vaccines at the prices assumed in this analysis, instead of having them donated, is likely to be challenging for lower-income countries. This result should be considered as a starting point to understanding the costs of maternal immunization programs in low- and middle-income countries.

## Introduction

Beginning in 2012, the World Health Organization (WHO) recommended that countries prioritize pregnant women above other high risk groups for receipt of influenza vaccine [1]. This recommendation was made in recognition of the elevated risk pregnant women have for severe influenza virus infection, as well as the programmatic opportunities the antenatal care (ANC) platform can provide for vaccine delivery [2]. Adoption of maternal influenza immunization has been slow, particularly in low resource settings [3,4]. To date, Gavi, the Vaccine Alliance has not supported maternal influenza immunization, substantially limiting the financial mechanisms to fund maternal influenza vaccine introduction in low resource countries.

National and international decision makers must consider both health and economic information, including program costs, when considering new vaccine introduction [5,6]. There is currently little economic evidence to inform maternal immunization programs in low- or middle-income settings though efforts are underway to remedy this need [7–9].

The purpose of this report is to describe the first evaluation of a Maternal Influenza Immunization Program Costing Tool (Costing Tool) for maternal immunization which was undertaken in Malawi. Malawi was selected for this pilot study due to its success in eliminating maternal and neonatal tetanus and to leverage related maternal influenza immunization feasibility studies that were being undertaken in-country. This is to our knowledge, the first costing study of maternal influenza immunization in a developing country.

## Methodology

### The costing tool

The Costing Tool is a Microsoft Excel-based micro-costing tool commissioned by WHO that estimates the incremental (additional) resources required to add maternal influenza vaccination to an existing national immunization program over a five-year period. It was developed as part of a series of economic tools to inform decision making and to plan the introduction of a maternal influenza immunization program [10–14]. The Costing Tool has its conceptual roots in the WHO Cervical Cancer Prevention and Control Costing (C4P) tool. The C4P tool was developed to help countries plan and project the cost of introducing cervical cancer interventions and has been widely used in Gavi-supported human papillomavirus (HPV) vaccine demonstration projects and scale up [15–19]. Similar to the C4P tool, the Costing Tool has been designed to help countries plan for maternal influenza immunization programs that may differ from infant vaccination because of differences in the target population and potential differences in delivery strategy or venue. For example, users can compare costs based on routine vaccination of pregnant women at fixed clinic sites and outreach sites to costs associated with campaigns. The tool also allows users to compare seasonal and year-round vaccination as well as national or regional vaccination or different coverage rates of scale-up. The Costing Tool estimates costs from the provider perspective and costs incurred by pregnant women or households are excluded.

The Costing Tool estimates costs for each of the major components of the immunization program as illustrated in Table 1. The user inputs data for each of these components in individual Excel worksheets or data collection forms developed for the Costing Tool. The tool then aggregates the costs incurred for each activity and categorizes them as introduction costs, recurrent costs, or capital costs. While the tool distinguishes capital and introduction costs, these categories are often grouped together in analyses since both last longer than a year.

**Table 1:** Introduction, recurrent, and capitals costs and their components.

Introduction (Start-up) Costs	Recurrent (Operational) Costs	Capital Costs
• Microplanning • Training • Social Mobilization and Information, Education, and Communication	• Vaccine and Injection Supplies • Service Delivery • Social Mobilization and Information, Education, and Communication • Supervision, Monitoring, and Evaluation • Other (e.g. waste management, items not included elsewhere)	• Cold chain equipment • Other equipment

In addition to these categories, the Costing Tool estimates initial investment costs which are upfront, non-annualized costs that include introduction costs with the addition of cold chain and other capital costs over the five-year time horizon of the Costing Tool. It is important to note that the Costing Tool annualizes introduction, cold chain, and other capital costs across the user defined “useful life years” but these are not annualized in the initial investment costs. However, if the useful life of a capital good exceeds the five-year horizon of the tool, only the share of the capital cost included in the five-year horizon will be captured. As such, initial investment costs are the best representation of the up-front cost of the vaccination program.

Across all of these categories, the Costing Tool utilizes and reports both financial and economic costs. Financial costs represent the value of resources purchased by the buyer such as the Ministry of Health (MOH). Financial costs are likely to include items such as the purchase of injection supplies, outreach allowances, and personnel per diems as well as purchased resources used in training and developing new communication materials. Economic costs represent the value of all resources used by the program regardless of who pays for those resources. Economic costs include all financial costs and are also likely to include items such as salaries of current health personnel, volunteer labor, donated supplies, and the opportunity cost of capital goods. The Costing Tool user guide contains a more comprehensive list of potential financial and economic costs by vaccination activity [14]. The user guide and S1 Appendix contain additional information on key vaccination activities and terms.

In addition to cost projections reported using the categories described above, the tool also reports several other cost metrics. The tool reports the number of immunized pregnant women on an annual basis, annual costs, and the cost per immunized woman across the time horizon of the tool. In this report, costs are reported in 2015 US Dollars, though the Costing Tool accommodates inputs and results in local currency or US Dollars for other reporting years.

The Costing Tool is designed to be adapted to the local context and incorporate country characteristics including demographics, administrative levels, and vaccination strategies (including delivery mode, coverage, and phased-in introduction). While it is important to adapt the tool to the local context, the tool generates the best results when local experts and an experienced facilitator collaborate to conduct the costing analysis.

### Data collection

In June 2015, the study team collected primary and secondary data on the costs of introducing a prospective maternal influenza immunization program after Malawi’s National Health Sciences Research Committee (NHSRC) provided Institutional Review Board approval for the study. PATH IRB deferred to NHSRC.

Qualitative and quantitative data were collected from primary and secondary sources at the national, zonal/regional, district, and facility levels. Key informant interviews were conducted at the National Expanded Program on Immunization (EPI) and Health Education Unit. Interviews were also conducted with program managers and at two health centers in each of three districts: Dowa, Rumphi and Zomba. Additional information was collected from follow up questions with the National EPI Logistician, the officer in charge of procurement at UNICEF Malawi, and the Infrastructure Development Planning Officer at the Ministry of Health. Information from secondary data sources (e.g. demographic health survey data) also supplemented the interviews.

Quantitative data on supply quantities, remuneration for staff time, expenditures, and other relevant expenses associated with vaccinations were gathered from record books at the national, district, and facility levels. These data were complemented by expert opinion in areas where documented information on the implementation of seasonal influenza vaccine was not available. Data collected included all costs linked to planning, training sessions at all administrative levels, social mobilization events, monitoring and supervision activities, procurement, storage of vaccines and supplies, immunization service delivery in static and outreach outlets, and eventual evaluation of the program.

### The Malawi costing tool pilot

The Costing Tool pilot in Malawi estimates the incremental cost of introducing a prospective maternal influenza vaccine. We assume delivery would be based on existing pediatric and ANC immunization infrastructure. This assumes a strong EPI and ANC program and coordination between the two programs. This study estimates both financial and economic costs from the government perspective over a five-year period beginning in 2018. Costs were collected in US Dollars and Malawian Kwacha. Kwacha were converted to US Dollars based on average UN Treasury Operational Rates between June 2014 and May 2015, i.e. the year prior to data collection. A start year of 2018 was selected as it represents costs within the relatively near term but would still allow a couple of years of preparation, if an introduction decision were made. For the purposes of this costing study, we assume Malawi would introduce seasonal maternal influenza immunization across all districts in the first year. Cost components are those illustrated in Table 1. Key data for the base scenario is provided in Table 2 with additional context in the following paragraphs and S1 Appendix. We also present alternative scenarios that vary some of the key cost drivers including ANC coverage, vaccine price, and presentation (e.g. number of doses per vial).

**Table 2:** Key data inputs, base scenario.

Input	Value	Source
**Target population (introduction year)**	913,000 pregnant women	Malawi NSO, Population projections 2008 [20,21]
**Doses per pregnant woman**	1	WHO Position Paper [1]
**ANC1 attendance**	95%	Demographic Health Survey [22]
**Vaccine coverage among ANC population**	74%	Assumption based on Demographic Health Survey data on other ANC services
**Vaccine price**	$0 financial cost due to assumed donation program; $2.9 economic cost	WHO Vaccine Product, Price and Procurement Database [23]
**Vaccine presentation**	Single dose pre-filled syringe	Assumption based on presentation used in current donation program
**Vaccine wastage**	5%	Assumption
**Vaccine buffer stock**	10%	Assumption
**Vaccine packaged volume**	60 cc	Reference volume by presentation in Immunization Financing Toolkit [24]
**Cold chain**	$7/liter for cold rooms; $26/liter for refrigerators	Malawi Cold Chain Assessment, 2011 [25]; Manufacturer Websites [25–34]; Project Optimize Analysis [31]
**Useful life years of cold chain equipment**	10	Interviews/assumption
**Vaccine transport**	Integrated into existing transport	Interviews/assumption
**Service delivery**	Three minute vaccinator time per woman in ANC clinic or outreach	Interviews
**Staff salaries**	Various	Interviews
**Microplanning, training, information, education, and communication**	Various	Interviews
**Supervision**	Various	Interviews
**Waste management**	Excluded	Assumption
**Disease surveillance**	Excluded	Assumption

We consider women who attend one ANC visits as a optimistic assumption for those that may receive vaccination in a low-income country setting. We begin with 95% of women who have at least one ANC visit as indicated by Malawi’s 2010 Demographic Health Survey and apply a uniform dropout rate between ANC visits one, two, three, and four [22]. We adjust this population downward to acknowledge that some women may not be offered or may not wish to receive the vaccine. We assume that Malawi has no excess cold chain capacity so expansion would be undertaken requiring additional cold rooms and refrigerators. No additional cold chain maintenance is included. Coverage would be achieved predominantly through ANC clinic visits, though some vaccination would occur during outreach. Microplanning, social mobilization, and supervision activities occur at various levels of the health system. Additional detail on the base scenario and data can be found in S1 Appendix.

The following section describes our base scenario results and then discusses alternative scenarios. Alternative scenarios include a lower ANC coverage scenario as well as a low and high coverage scenario in which Malawi would purchase the vaccine in a 10 dose vial.

## Results

### Scenario-base

#### Financial and economic cost of the immunization program

The base scenario financial cost of the maternal immunization program is approximately $1.2 million over five years. Overall financial and economic costs are displayed in Table 3 below. While most of the discussion below focuses on financial costs, cost categories that include labor or donated resources (e.g. microplanning, training, social mobilization, service delivery, vaccines and supplies) will be substantially higher from an economic cost perspective. Because our base scenario assumes a donated vaccine, the $8.5 million vaccine cost is only included as an economic cost of the program.

**Table 3:** Financial and economic costs of maternal influenza immunization program, base scenario.

Activity		Financial Costs 2018–2022, USD (% of total)	Economic Costs 2018–2022, USD (% of total)
Introduction		788,584 (68)	1,399,276 (13)
	Microplanning	159,811 (14)	304,011 (3)
	Training	150,790 (13)	460,705 (4)
	Social Mobilization/IEC	76,938 (7)	176,247 (2)
	Cold Chain Supplementation	401,045 (34)	458,313 (4)
Recurrent		383,308 (34)	8,982,402 (88)
	Continuing IEC	55,192 (5)	55,192 (1)
	Service Delivery	184,225 (16)	277,406 (3)
	Vaccines and Supplies	12,505 (1)	8,504,410 (82)
	Supervision Monitoring Evaluation	112,691 (10)	126,687 (1)
	Other Recurrent Costs	17,775 (2)	17,987 (1)
Total Costs		1,170,974	10,380,958

Over the five-year time period, introduction costs account for approximately 70% of financial costs with recurrent costs accounting for approximately 30%. Introduction costs include microplanning, training and social mobilization/information, education, and communication (IEC) at 14%, 13%, and 7% respectively. Capital costs, i.e. cold chain, account for 34% of total financial costs. Among recurrent costs, service delivery and supervision account for 16% and 10% of total costs respectively. The bulk of service delivery costs are associated with outreach (i.e. per diem and transport). Continuing IEC contributes 5% of total costs. Vaccines and supplies are only a small component of financial costs as this scenario assumes a donated vaccine; other recurrent costs are minimal.

**Fig 1:**
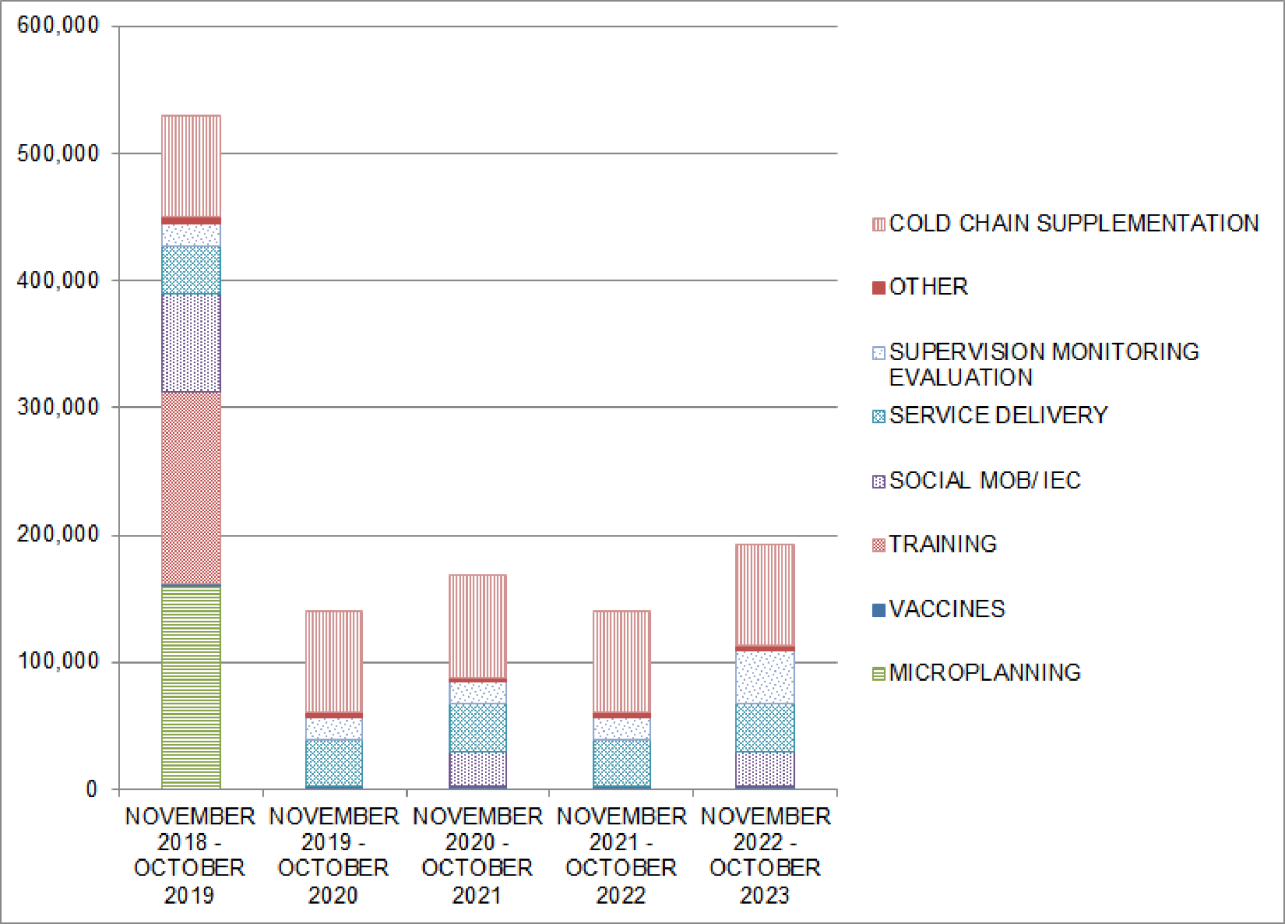
Financial costs of maternal influenza immunization program by year, (US$).

We now examine the annual costs of the program over five years. Fig 1 demonstrates several important points. First, financial costs in the first year are much higher (more than $500,000) than costs in the ensuing years (approximately $150,000 to $200,000), because most introduction costs are incurred in year one. Second, costs in the introduction year are widely distributed across categories.

Microplanning, training, and social mobilization are the largest categories while cold chain, service delivery, and supervision are smaller cost components. In the ensuing years, cold chain, service delivery, and supervision are the main costs but social mobilization adds costs every two years. Notably, vaccines and supplies are only a small financial cost since we assume a donated vaccine.

From an economic cost perspective (S1 Fig), the cost of the vaccine is substantially greater than all other cost categories over the life of the program when the vaccine is valued at $2.90 per dose. However, training, microplanning, and social mobilization are substantial costs in year one.

Over the initial five years of the immunization program, 2.3 million pregnant women would be reached under our base scenario assumptions. Over this period, the cost per immunized pregnancy is estimated at $0.52 (financial) and $4.58 (economic). Immunization coverage is 47% of the eligible population.

### Other scenarios

The base scenario described above represents the costs associated with a potential maternal influenza immunization program in a low-income country. We now examine a scenario with a less optimistic ANC coverage and then examine the costs of a maternal influenza immunization program with a purchased vaccine and different presentation, under both low and higher coverage scenarios.

#### Scenario-base with lower vaccine coverage

The second scenario replicates the base scenario but decreases vaccine coverage. This scenario assumes that women with at least four ANC visits will be eligible to receive influenza vaccine if they attend during the vaccination season. It starts with 46% of women who have at least four ANC visits as indicated by Malawi’s 2010 Demographic Health Survey but assume no dropoutsbetween ANC visits one, two, three, and four [22]. We again assume that 74% of the women that attend ANC visits will receive vaccination based on the average coverage of other ANC services in Malawi [22].

Overall financial and economic costs decrease to $1.0 and $6.3 million, respectively. The financial and economic cost per immunized pregnant woman falls to $0.79 and $5.06, respectively. The higherer cost per immunized woman is due to spreading fixed costs over 1.1 million fewer immunized pregnant women. Immunization coverage is 26% of the eligible population. Additional detail on this scenario can be found in Table A in S1 Appendix, S2 Fig and S3 Fig.

#### Scenario-purchased vaccine, 10 dose vial, low coverage

This scenario considers only women that attend four or more ANC visits as the potential beneficiaries. Malawi purchases a vaccine rather than benefits from a donation program. Since no low- or lower-middle income countries reported purchasing a 10 dose vial of seasonal influenza vaccine in the WHO Vaccine Product, Price and Procurement Database, we utilized the lowest reported price ($2.80) for a 10 dose vial reported by Pan American Health Organization [23]. The change in presentation required an administering syringe ($0.05) and increased vaccine wastage to 40%. The 10 dose vial reduces vaccine volume (3 cc) and cold chain costs [23,24]. Cold chain costs were calculated as in the base scenario but are lower due to the decrease in vaccine volume.

Purchasing a vaccine greatly increases the financial cost of vaccines and supplies but reduces cold chain storage requirements and costs (see Table 4 below). As in the low coverage scenario, 1.2 million pregnant women would be immunized, but the financial (economic) cost would be $6.23 ($6.73) per immunized pregnant woman. Immunization coverage is 26% of the eligible population. Annual program costs are available in S4 Fig and S5 Fig.

**Table 4:** Financial and economic costs of maternal influenza immunization program, purchased vaccine in 10 dose vial (low and high coverage scenarios).

		Low Coverage		High Coverage	
Activity		Financial Costs 2018–2022, USD (% of total)	Economic Costs 2018–2022, USD (% of total)	Financial Costs 2018–2022, USD (% of total)	Economic Costs 2018–2022, USD (% of total)
Introduction		404,343 (6)	960,167 (13)	418,273 (4)	976,086 (7)
	Microplanning	159,811 (2)	304,011 (4)	159,811 (1)	304,011 (2)
	Training	150,790 (2)	460,705 (6)	150,790 (1)	460,705 (3)
	Social mobilization/IEC	76,938 (1)	176,247 (2)	76,938 (1)	176,247 (1)
	Cold Chain Supplementation	16,804 (1)	19,204 (1)	30,734 (1)	35,123 (1)
Recurrent		7,316,078 (96)	7,381,233 (90)	13,074,374 (98)	13,181,763 (95)
	Continuing IEC	55,192 (1)	55,192 (1)	55,192 (1)	55,192 (1)
	Service Delivery	184,225 (2)	235,172 (3)	184,225 (1)	277,406 (2)
	Vaccines and Supplies	6,946,195 (90)	6,946,195 (83)	12,704,491 (94)	12,704,491 (90)
	Supervision Monitoring and Evaluation	112,691 (2)	126,687 (2)	112,691 (1)	126,687 (1)
	Other Recurrent Costs	17,775 (1)	17,987 (1)	17,775 (1)	17,987 (1)
Total Costs		7,720,423	8,341,400	13,492,648	14,137,849

#### Scenario-purchased vaccine, 10 dose vial, high coverage

This scenario returns to high vaccine coverage with 95% of women who having at least one ANC visit and a uniform dropout rate between ANC visits. The overall cost of the program increases to $13.5 million over five years, primarily due to vaccine and supply costs. The program reaches 2.3 million women at a financial (economic) cost per immunized pregnant woman of $5.95 ($6.24). Immunization coverage is 47% of the eligible population. Annual program costs are available in S6 Fig and S7 Fig.

## Discussion

In this costing study of a maternal influenza immunization program in Malawi, base scenario financial and economic costs per dose delivered are approximately $0.50 and $4.50 respectively. Introduction costs for our base scenario account for 70% of total financial costs across the five-year period and recurrent costs are the remaining 30% of total financial costs. Microplanning, training, and cold chain are the largest components of introduction costs. Service delivery is the largest component of recurrent costs at 16% of total financial costs. Relative to some new vaccine introductions such as HPV, costs associated with social mobilization are slightly lower but in the same range. For example, Hutubessy et al. (2012) find that social mobilization and IEC account for 10% of the financial costs [15]. Our base scenario estimates 7% of financial costs in this category. However, we are excluding the cost of the vaccine and when this is incorporated into our analysis, social mobilization and IEC only account for 1% to 3% of costs depending on the scenario. While social mobilization and IEC are important components of new vaccine introductions, slightly lower costs may be reasonable in this case as many women are already attending ANC and such a program would not always necessitate an additional visit.

Our base scenario includes vaccine costs only as an economic cost. With this in mind, our base scenario financial cost per dose roughly corresponds to recent multicountry analyses of immunization delivery costs [35,36]. Average annual financial and economic costs over five years, achieving 47% coverage of the eligible population, are approximately $240,000 and $2.1 million respectively. We are unaware of other studies that use primary data to estimate delivery costs for vaccine programs for pregnant women in developing countries. Among analyses of childhood immunization programs, there is wide variation in immunization delivery cost estimates [37]. Some newer studies find delivery costs to be several times greater than our analysis reflects [38–40]. Our findings are not unexpected given that we are estimating the cost of introducing a new vaccine program while assuming much of the immunization and ANC system can serve as a basis for a maternal immunization program. This is an optimistic but not implausible basis for this analysis and our results should be interpreted in this context.

Noting the assumptions made in our base scenario, delivery costs do not seem overwhelming. However, if Malawi or another low-income country were to consider purchasing a vaccine, this would change the financial outlook. For example, purchasing a vaccine at the lowest prices reported by a low- or lower- middle income country increases the financial costs per dose to between $5 and $7 (depending on the scenario) and would have dramatic budget implications. While a full assessment of affordability is beyond the scope of this costing analysis, we note that Malawi’s per capita total health expenditure is $26 per year meaning that the budget impact of a maternal influenza immunization program would be large [21]. Each option should be carefully considered in terms of the health and economic benefit, costs, available resources, and the cost-effectiveness of this and alternative uses of resources.Recent publications have explored the cost-effectiveness of maternal influenza immunization in lower-income settings using values broadly similar to the findings on this study. Further empirical costing evidence on maternal influenza immunization, potentially generated through the use of this tool, can enhance and help validate the findings of those studies [41]. The case for maternal influenza immunization becomes more difficult absent support from international donors.

Program costs and benefits are not the only consideration [6]. Introducing a maternal influenza immunization program would also test the assumption that the current immunization and ANC systems are able to absorb a new immunization program. While immunization (DTP3) coverage has been hovering around 90% in Malawi [42], it is important to consider whether a new program would lead to reduced coverage for other antigens. The same question must also be asked of the ANC program. Per the Demographic Health Survey, many ANC attendees do not receive all ANC services [22]. If an additional service (e.g. maternal influenza vaccination) were offered, would this have negative consequences on other components of ANC? Conversely, maternal influenza vaccination might also encourage higher attendance and lead to additional health benefit beyond influenza prevention. This brief discussion is not meant to highlight all of the potential challenges or opportunities in introducing maternal immunization programs in low-income countries. However, it does highlight the importance of understanding the factors that may inhibit or facilitate success. Some work has been done on this important topic, but additional research is needed [43].

## Limitations

There are several limitations to this study that deserve mention. First, this study was used to pilot a new tool that is not yet WHO endorsed. All results should be considered in relation to current knowledge and future piloting. In addition, both data collection and analysis were affected by the uncertainty surrounding a prospective immunization program so our interviews with EPI program staff were not informed by active maternal immunization program planning. This analysis also assumes many activities are built upon a well-functioning system so a number of activities would not incur incremental costs (e.g. vaccine transport, waste management, or much outreach). While we believe our estimates are realistic, they are only reflective of the delivery strategy and our knowledge of the country context. If a country selected a strategy that required more stand-alone outreach or supplemental activities, costs would increase. The Costing Tool does not allow for uncertainty analysis outside of examining alternative scenarios so these scenarios should be interpreted carefully. Finally, the costs of EPI/ANC program coordination are not included in these estimates and would increase the costs of the program slightly.

## Conclusion

This study demonstrates the successful use of a new influenza vaccine costing tool and estimates the financial and economic costs associated with a prospective maternal influenza immunization program in a low-income country. The information gained through this study not only helps inform our understanding of costs in Malawi, but may also benefit other countries as little is known of the costs of maternal immunization programs. Our analysis estimates that the incremental cost of a maternal influenza immunization program integrated into the ANC platform does not differ dramatically from the incremental costs one might expect if adding another antigen to a childhood vaccination program on a cost per dose basis. Maternal immunization delivery costs may be reasonable assuming the EPI and ANC system is capable of serving as the platform for an additional vaccination program. However, purchasing influenza vaccines at the prices assumed in this analysis is likely to be challenging for lower-income countries. These conclusions should be viewed as the product of one of the first studies of its kind and should be interpreted cautiously until further corroboration. WHO will conduct multiple iterations of piloting and refinement of the tool with eventual plans for a formal review by immunization economics experts and endorsement by WHO IVIR-AC before the tool is made available in the public domain. While we believe our results accurately reflect costs under the scenarios described, these findings should be corroborated by similar studies in other countries to confirm their generalizability and applicability to the local context prior to being used for policy decisions.

## Supporting information

S1 Fig Economic costs of maternal influenza immunization program by year (base scenario), (US$). (TIF)

S2 Fig Financial costs of maternal influenza immunization program by year (base scenario with lower ANC coverage), (US$). (TIF)

S3 Fig Economic costs of maternal influenza immunization program by year (base scenario with lower ANC coverage), (US$). (TIF)

S4 Fig Financial costs of maternal influenza immunization program by year (purchased vaccine, 10 dose vial, low coverage), (US$). (TIF)

S5 Fig Economic costs of maternal influenza immunization program by year (purchased vaccine, 10 dose vial, low coverage), (US$). (TIF)

S6 Fig Financial costs of maternal influenza immunization program by year (purchased vaccine, 10 dose vial, high coverage), (US$). (TIF)

S7 Fig Economic costs of maternal influenza immunization program by year (purchased vaccine, 10 dose vial, high coverage), (US$). (TIF)

S1 File Costing tool, Malawi base scenario. (XLSX)

S1 Appendix (DOCX)

## References

[1] WHO. Vaccines against influenza WHO position paper—November 2012. Wkly Epidemiol Rec. 2012;87: 461–476. 23210147

[2] WHO. Meeting of the strategic advisory group of experts on immunization, April 2015: conclusions and recommendations. Wkly Epidemiol Rec. 2015;90: 261–280. 26027016

[3] Keller-Stanislawski B, Englund JA, Kang G, Mangtani P, Neuzil K, Nohynek H, et al Safety of immunization during pregnancy: a review of the evidence of selected inactivated and live attenuated vaccines. Vaccine. 2014;32: 7057–7064. doi: 10.1016/j.vaccine.2014.09.052 25285883 10.1016/j.vaccine.2014.09.052

[4] Ortiz JR, Perut M, Dumolard L, Wijesinghe PR, Jorgensen P, Ropero AM, et al A global review of national influenza immunization policies: Analysis of the 2014 WHO/UNICEF Joint Reporting Form on immunization. Vaccine. 2016;34: 5400–5405. doi: 10.1016/j.vaccine.2016.07.045 27646030 10.1016/j.vaccine.2016.07.045 PMC5357765

[5] Abramson JS, Mason E. Strengthening maternal immunisation to improve the health of mothers and infants. Lancet. 2016;388: 2562–2564. doi: 10.1016/S0140-6736(16)30882-0 27372397 10.1016/S0140-6736(16)30882-0

[6] WHO. How to implement influenza vaccination of pregnant women: an introduction manual for national immunization programme managers and policy makers Geneva, Switzerland: World Health Organization; 2016 http://apps.who.int/iris/bitstream/10665/250084/1/WHO-IVB-16.06-eng.pdf

[7] Peasah SK, Azziz-Baumgartner E, Breese J, Meltzer MI, Widdowson MA. Influenza cost and cost-effectiveness studies globally—a review. Vaccine. 2013;31: 5339–5348. doi: 10.1016/j.vaccine.2013.09.013 24055351 10.1016/j.vaccine.2013.09.013

[8] Ott JJ, Klein BJ, Tam JS, Hutubessy RC, Jit M, de Boer MR. Influenza vaccines in low and middle income countries: a systematic review of economic evaluations. Hum Vaccin Immunother. 2013;9: 1500–1511. doi: 10.4161/hv.24704 23732900 10.4161/hv.24704 PMC3890229

[9] de Francisco SN, Donadel M, Jit M, Hutubessy R. A systematic review of the social and economic burden of influenza in low- and middle-income countries. Vaccine. 2015;33: 6537–6544. doi: 10.1016/j.vaccine.2015.10.066 26597032 10.1016/j.vaccine.2015.10.066

[10] WHO. A manual for estimating disease burden associated with seasonal influenza Geneva, Switzerland: World Health Organization; 2015 http://apps.who.int/iris/bitstream/10665/178801/1/9789241549301_eng.pdf?ua=1

[11] WHO. Guidance on the economic evaluation of influenza vaccination Geneva, Switzerland: World Health Organization; 2016 http://www.who.int/immunization/research/development/influenza_maternal_immunization/en/index2.html

[12] WHO. Tool for estimating the economic burden associated with seasonal influenza Geneva, Switzerland: World Health Organization; 2016 http://www.who.int/entity/immunization/research/development/Economic_burden_Toolkit.xlsx?ua=1

[13] WHO. WHO manual for estimating the economic burden of seasonal influenza Geneva, Switzerland: World Health Organization; 2016 http://www.who.int/immunization/research/development/influenza_maternal_immunization/en/index2.htm

[14] WHO. WHO Flutool for planning and costing maternal influenza vaccination: pilot version 1.0 Geneva, Switzerland: World Health Organization; 2016 http://apps.who.int/iris/bitstream/10665/250087/1/WHO-IVB-16.07-eng.pdf?ua=1

[15] Hutubessy R, Levin A, Wang S, Morgan W, Ally M, John T, et al A case study using the United Republic of Tanzania: costing nationwide HPV vaccine delivery using the WHO Cervical Cancer Prevention and Control Costing Tool. BMC Med. 2012;10: 136 doi: 10.1186/1741-7015-10-136 23146319 10.1186/1741-7015-10-136 PMC3520749

[16] Levin A, Wang SA, Levin C, Tsu V, Hutubessy R. Costs of introducing and delivering HPV vaccines in low and lower middle income countries: inputs for GAVI policy on introduction grant support to countries. PLoS One. 2014;9: e101114 doi: 10.1371/journal.pone.0101114 24968002 10.1371/journal.pone.0101114 PMC4072768

[17] Ngabo F, Levin A, Wang SA, Gatera M, Rugambwa C, Kayonga C, et al A cost comparison of introducing and delivering pneumococcal, rotavirus and human papillomavirus vaccines in Rwanda. Vaccine. 2015;33: 7357–7363. doi: 10.1016/j.vaccine.2015.10.022 26519548 10.1016/j.vaccine.2015.10.022 PMC5357722

[18] Levin CE, Van MH, Odaga J, Rout SS, Ngoc DN, Menezes L, et al Delivery cost of human papillomavirus vaccination of young adolescent girls in Peru, Uganda and Viet Nam. Bull World Health Organ. 2013;91: 585–592. doi: 10.2471/BLT.12.113837 23940406 10.2471/BLT.12.113837 PMC3738308

[19] WHO. WHO cervical cancer prevention and control costing tool (C4P) user's guide: version 1.0 Geneva, Switzerland: World Health Organization; 2012 http://www.who.int/immunization/diseases/hpv/cervical_cancer_costing_tool/en/

[20] Malawi NSO. Population projections Malawi Zomba, Malawi: National Statistical Office of Malawi; 2008 http://www.nsomalawi.mw/images/stories/data_on_line/demography/census_2008/Main%20Report/ThematicReports/Population%20Projections%20Malawi.pdf

[21] World Bank. World development indicators Washington, DC, USA: International Bank for Reconstruction and Development/The World Bank; 2015 http://data.worldbank.org/products/wdi

[22] National Statistical Office. Malawi demographic and health survey 2010: final report. Calverton, MD, USA: ICF Macro; 2011. http://apps.who.int/iris/bitstream/10665/250087/1/WHO-IVB-16.07-eng.pdf?ua=1

[23] WHO. Vaccine product, price and procurement (V3P) database Geneva, Switzerland: World Health Organization; 2015 http://apps.who.int/immunization/vaccineprice/en/Navigation/Load?menu=1100

[24] World Bank and GAVI Alliance. Immunization financing toolkit; a resource for policy-makers and program managers Washington, DC, USA: World Bank; 2010 http://siteresources.worldbank.org/HEALTHNUTRITIONANDPOPULATION/Resources/281627-1292531888900/IMMUNIZATIONFINANCINGTOOLKITFINAL121410.pdf

[25] Malawi Ministry of Health. Cold chain assessment: inventory of cold chain equipment and assessment of capacity requirements from 2011–2015 Lilongwe, Malawi: Government of Malawi; 2011.

[26] Technology Exchange Lab, Inc. Cambridge, MA, USA: Cambridge Innovation Center; 2016. http://www.techxlab.org/

[27] Icelined refrigerators. Esbjerg, Denmark: Vestfrost Solutions; 2016. http://www.vestfrostsolutions.com/icelined-refrigerators/

[28] Performance quality safety (PQS) catalogue: prequalified devices and equipment—E003 refrigerators and freezers for storing vaccines and freezing waterpacks (E003/012 Icelined refrigerator, Vestfrost Solutions, MK 404) Geneva, Switzerland: World Health Organization; 2010 http://www.who.int/immunization_standards/vaccine_quality/pqs_e003_012_vestfrost_mk404.pdf

[29] ICRC. Emergency medical items catalogue: refrigerator 170L (55L vaccines)/freezer, Sibir V170 Geneva, Switzerland: International Federation of Red Cross and Red Crescent Societies; 2011 http://procurement.ifrc.org/catalogue/detail.aspx?volume=2&groupcode=204&familycode=204001&categorycode=FRIF&productcode=XCOLFRIF05

[30] Solar refrigerators. Esbjerg, Denmark: Vestfrost Solutions; 2016. http://www.vestfrostsolutions.com/solar-refrigerators/

[31] PATH. Vaccine regional distribution center cost assessment Seattle, WA, USA: PATH; 2011 http://www.path.org/publications/files/TS_opt_rdc_rpt.pdf

[32] WHO. Effective vaccine management (EVM) initiative Geneva, Switzerland: World Health Organization; 2016 http://www.who.int/immunization/programmes_systems/supply_chain/evm/en/index3.html

[33] WHO. Influenza vaccine (seasonal)—inactivated (10 dose vial) Geneva, Switzerland: World Health Organization; 2015 http://www.who.int/immunization_standards/vaccine_quality/pq_239_influenza_seasonal_10dose_sanofi_pasteur/en/

[34] Malawi Expanded Programme on Immunisation. Malawi comprehensive EPI multi-year plan 2012–2016 Lilongwe, Malawi: Government of Malawi; 2011.

[35] Brenzel L. What have we learned on costs and financing of routine immunization from the comprehensive multi-year plans in GAVI eligible countries? Vaccine. 2015;33 Suppl 1: A93–A98. 25919183 10.1016/j.vaccine.2014.12.076

[36] Galactionova K, Bertram M, Lauer J, Tediosi F. Costing RTS,S introduction in Burkina Faso, Ghana, Kenya, Senegal, Tanzania, and Uganda: A generalizable approach drawing on publicly available data. Vaccine. 2015;33: 6710–6718. doi: 10.1016/j.vaccine.2015.10.079 26518406 10.1016/j.vaccine.2015.10.079 PMC5357730

[37] Levin C. Convening on immunization delivery costs: meeting report. Seattle, WA, USA: Department of Global Health, University of Washington; 2011. http://static1.squarespace.com/static/556deb8ee4b08a534b8360e7/t/56cfda5d356fb0b23f34b5ae/1456462429679/FINAL+Delivery+Cost+meeting+report.pdf

[38] Le Gargasson JB, Nyonator FK, Adibo M, Gessner BD, Colombini A. Costs of routine immunization and the introduction of new and underutilized vaccines in Ghana. Vaccine. 2015;33 Suppl 1: A40–A46. 25919173 10.1016/j.vaccine.2014.12.081

[39] Schutte C, Chansa C, Marinda E, Guthrie TA, Banda S, Nombewu Z, et al Cost analysis of routine immunisation in Zambia. Vaccine. 2015;33 Suppl 1: A47–A52. 25919174 10.1016/j.vaccine.2014.12.040

[40] Janusz CB, Castaneda-Orjuela C, Molina A, I, Felix Garcia AG, Mendoza L, Diaz IY, et al Examining the cost of delivering routine immunization in Honduras. Vaccine. 2015;33 Suppl 1: A53–A59. 25919175 10.1016/j.vaccine.2015.01.016

[41] Orenstein E, Be Orenstein L, Diarra K, Djiteye M, Sidibe D, Haidara F, et al Cost-effectiveness of maternal influenza immunization in Bamako, Mali: A decision analysis. PLoS ONE. 2017;12: 1–16. 10.1371/journal.pone.0171499 PMC5295679 28170416

[42] Gavi (2016) Malawi (country hub). Gavi Available: http://www.gavi.org/country/malawi/. Accessed: 14 December 2016.

[43] PATH. Maternal influenza immunization: perceptions of decision-makers, healthcare providers, and the community in Malawi (unpublished; report in preparation) Seattle, WA, USA: PATH; 2016.

