# Supplementary figures and images for: Maternal influenza immunization in Malawi: Piloting a maternal influenza immunization program costing tool by examining a prospective program
Source: PLoS One. 2017 Dec 27;12(12):e0190006. doi: 10.1371/journal.pone.0190006 (PMC5744963; doi:10.1371/journal.pone.0190006)

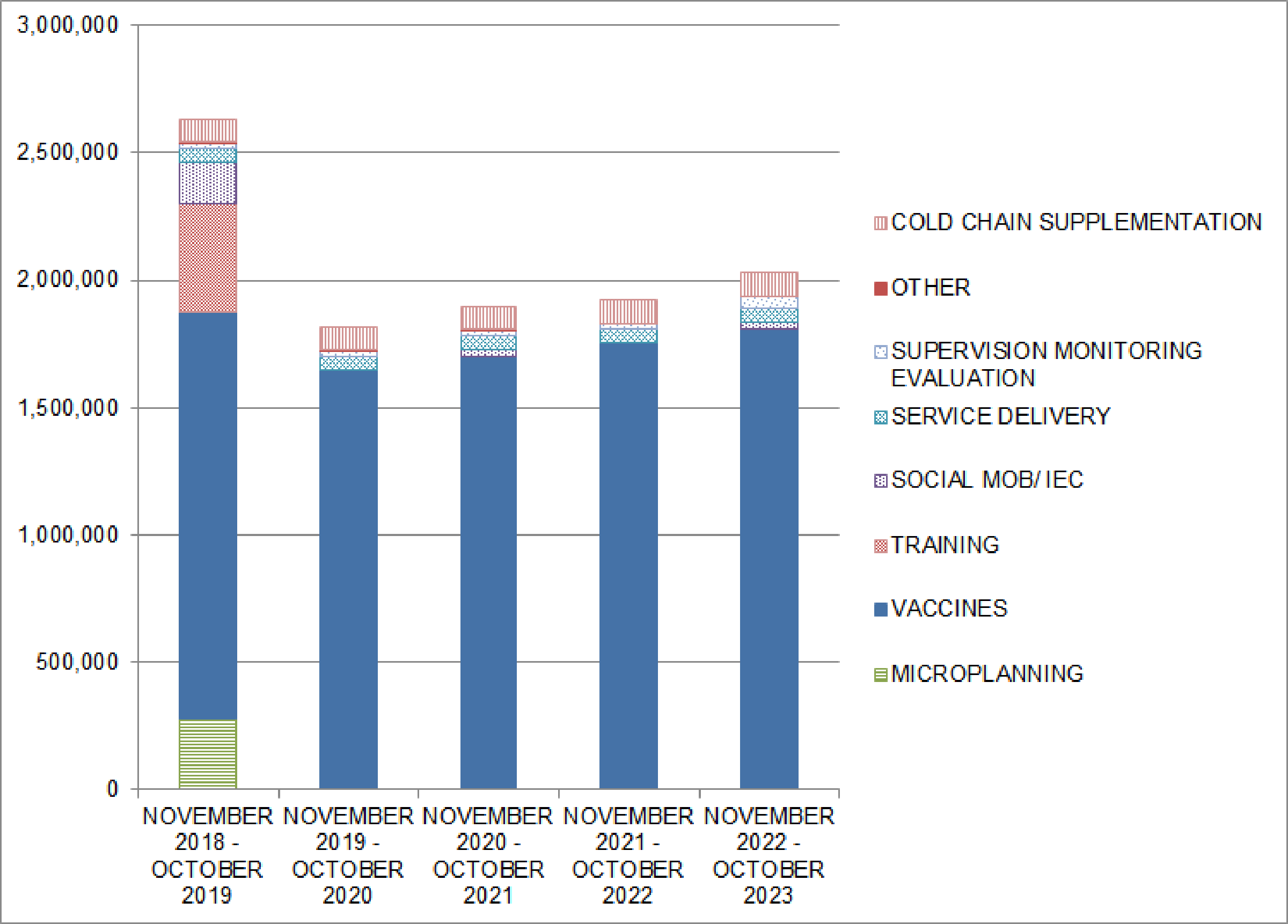

Supplement: S1 Fig — (TIF) [file pone.0190006.s001.tif]

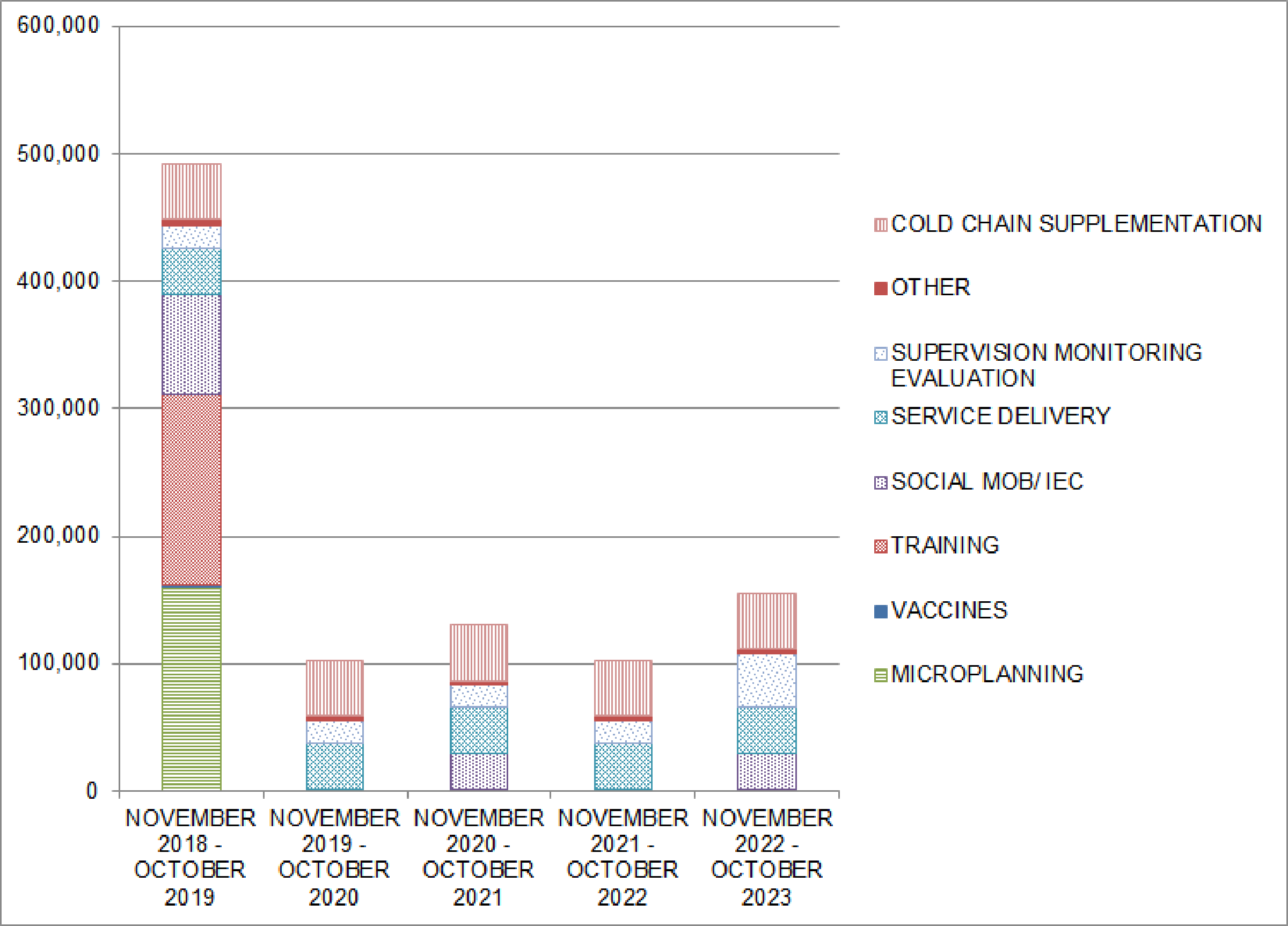

Supplement: S2 Fig — (TIF) [file pone.0190006.s002.tif]

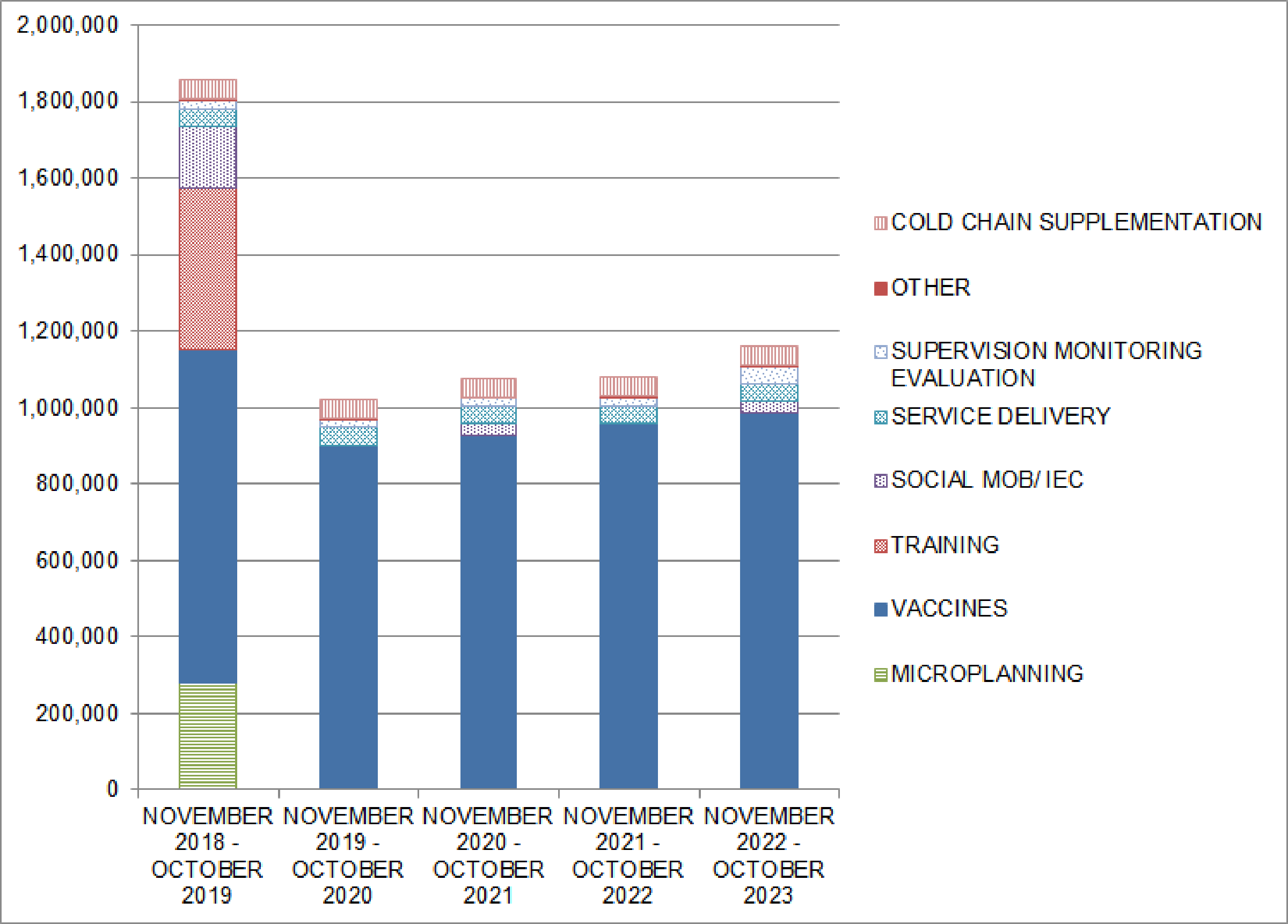

Supplement: S3 Fig — (TIF) [file pone.0190006.s003.tif]

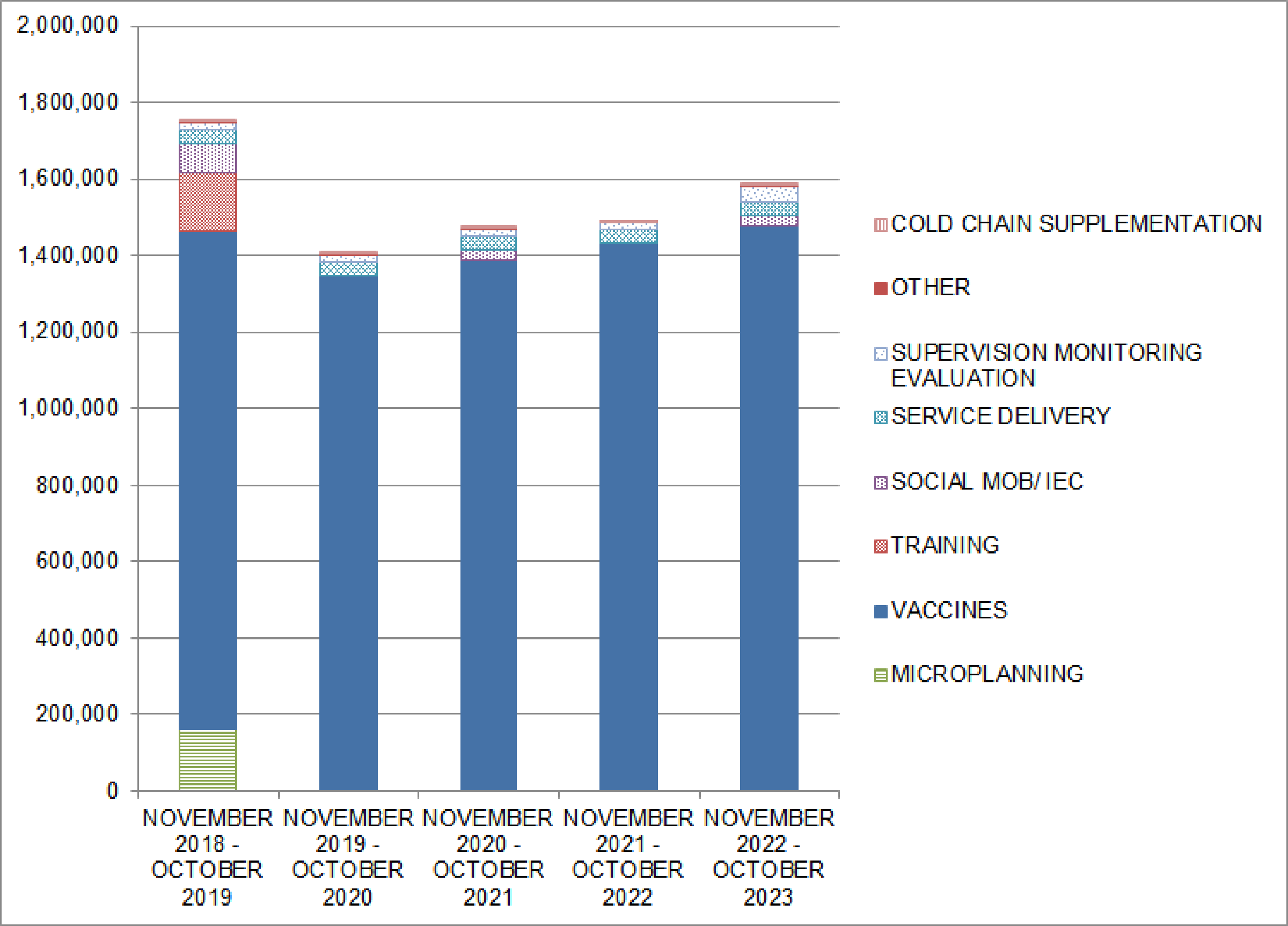

Supplement: S4 Fig — (TIF) [file pone.0190006.s004.tif]

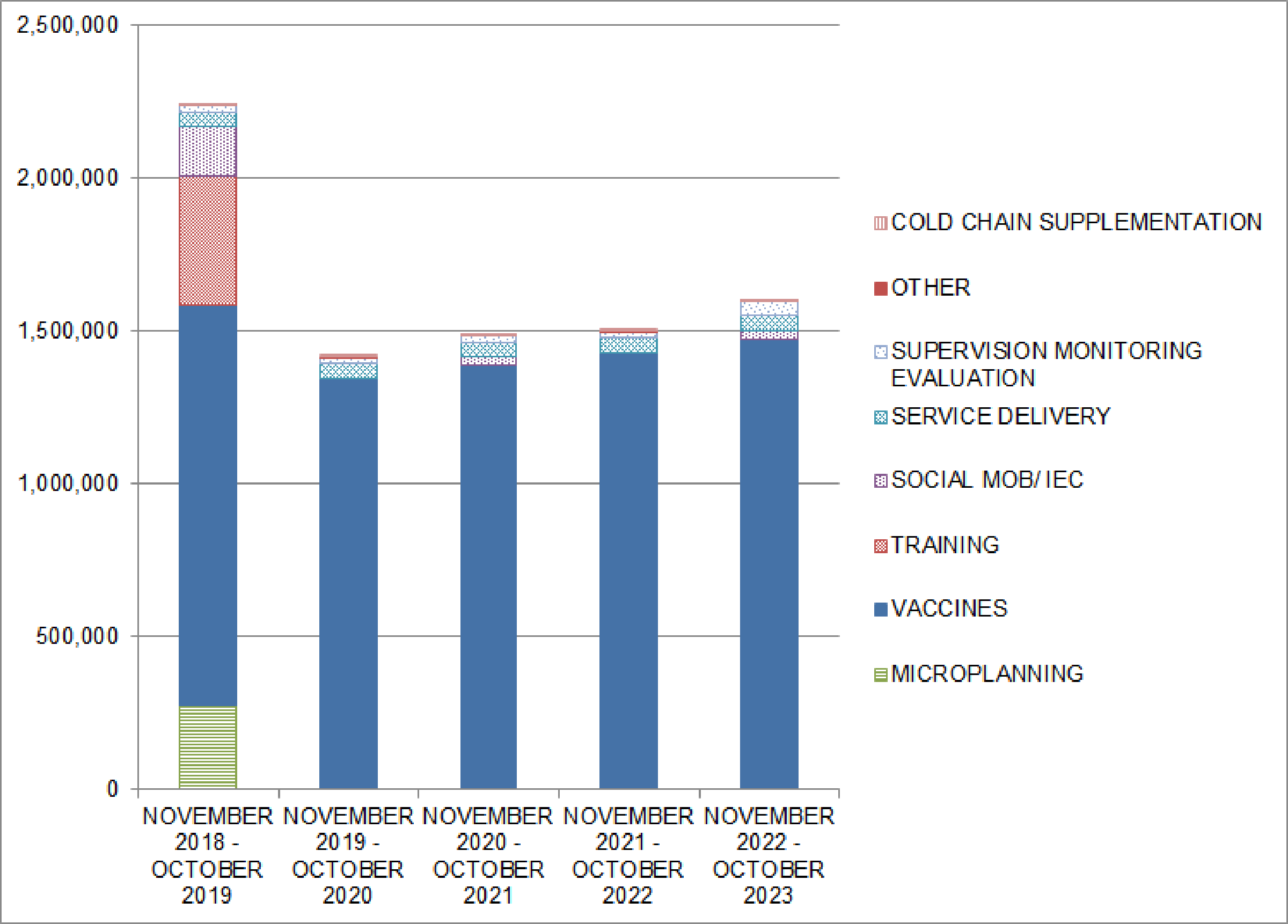

Supplement: S5 Fig — (TIF) [file pone.0190006.s005.tif]

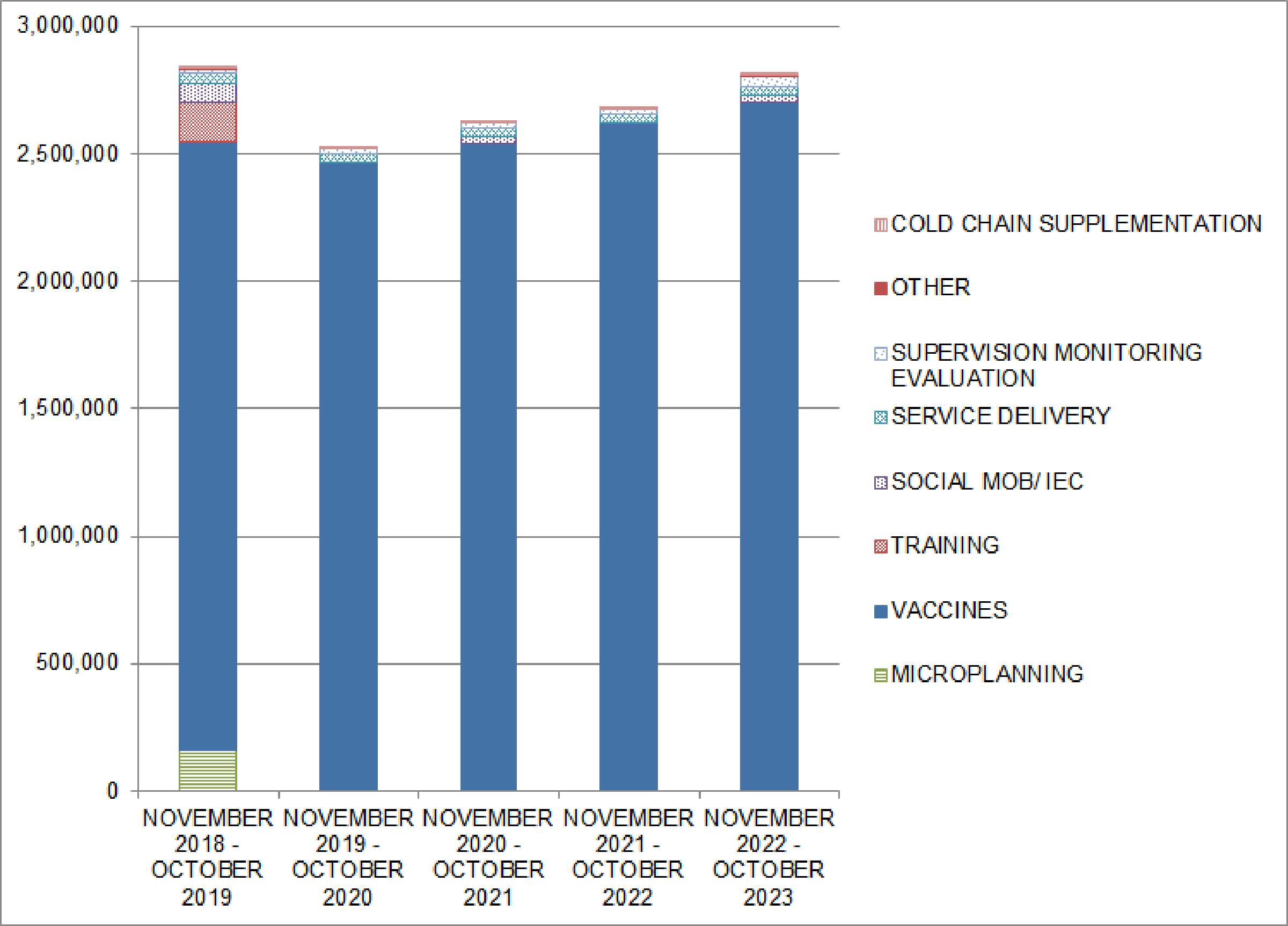

Supplement: S6 Fig — (TIF) [file pone.0190006.s006.tif]

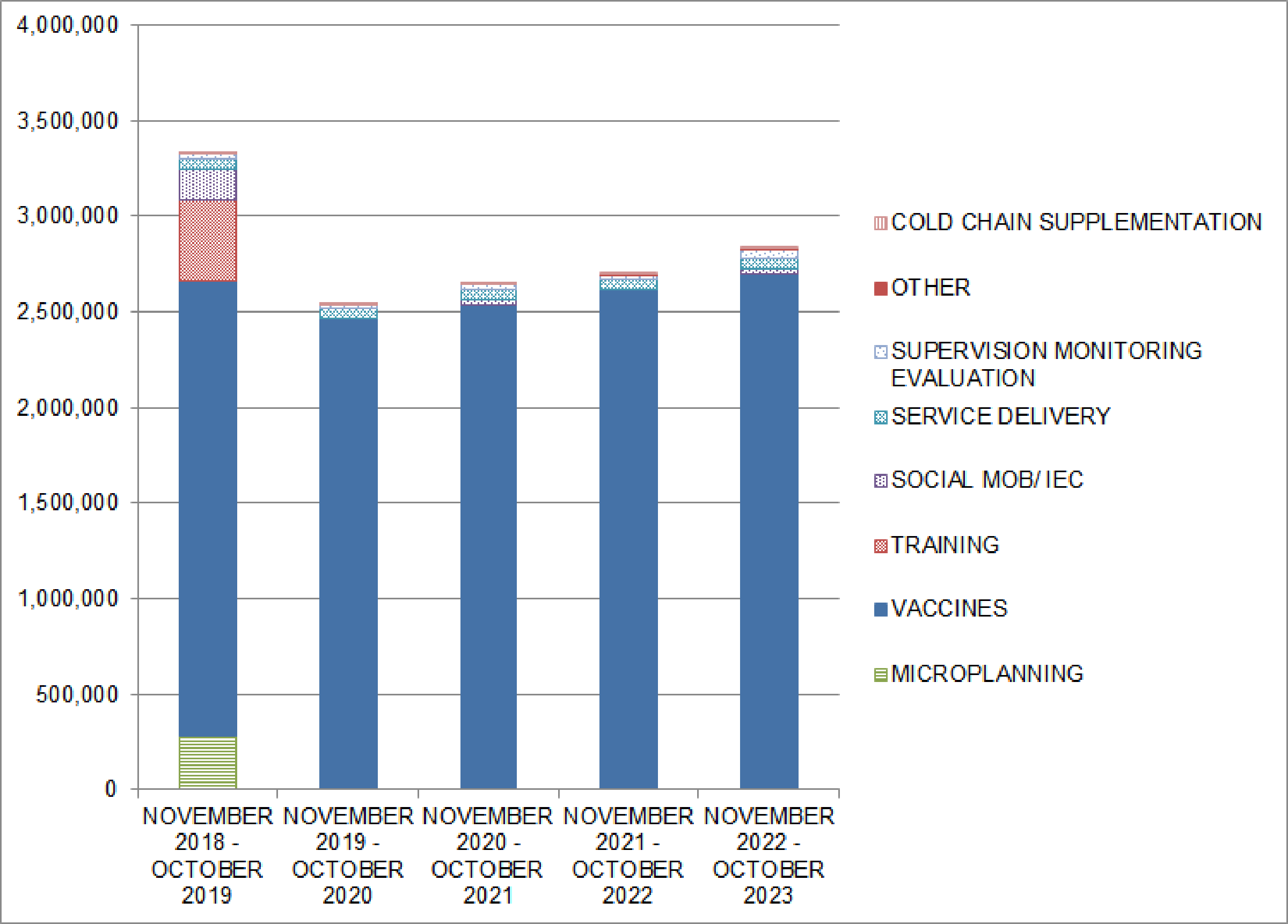

Supplement: S7 Fig — (TIF) [file pone.0190006.s007.tif]
