# Supplementary material for: Maternal influenza immunization in Malawi: Piloting a maternal influenza immunization program costing tool by examining a prospective program
Source: PLoS One. 2017 Dec 27;12(12):e0190006. doi: 10.1371/journal.pone.0190006 (PMC5744963; doi:10.1371/journal.pone.0190006)
Supplement: S1 Appendix — (DOCX) [file pone.0190006.s009.docx]

**Appendix**

**Guide to key terms**

Buffer stock-A safety stock that serves as a cushion against emergencies, major fluctuations in vaccine demands or unexpected transport delays [1].

Cold chain-The entire chain of storage facilities and transportation links through which supplies move from manufacturer to consumer, including port facilities, the primary store, intermediate stores, all service delivery points, equipment and transport vehicles [2].

Microplanning-A detailed set of plans across the various levels of the immunization system to guide the preparation, introduction and delivery of a vaccine.

Monitoring book-A book or books to record key immunization programs metric, most importantly vaccine coverage.

Outreach-Vaccination activities that occur outside of fixed facilities. Outreach activities often require immunization program staff to travel to reach beneficiaries.

Social mobilization-A broad set of messages and materials that, alongside other advocacy and communications efforts, are designed to educate and influence the behavior of a broad range of stakeholders including decision makers, community members and health workers.

Wastage-A measure of the share of a commodity that is not used due to expiration, breakage or other factors. For vaccines, it is calculated as 1 - (doses delivered/doses issued).

**Supplemental methodological background and data inputs**

The vaccination program targets a total of 913,000 pregnant women in 2018. This population increases at 3.1% per year to account for population growth [3,4]. Our base scenario assumes a seasonal vaccination program that occurs during six months of the year based on a primary influenza season of February through April plus the three preceding months [5]. While the Flu Costing Tool assumes a uniform distribution of pregnancies across the year, pregnant women are only vaccinated during these six months. Per Malawi’s 2010 Demographic and Health Survey (DHS), 95% of pregnant women attend a least one ANC visit, and 46% of pregnant women attend four or more ANC visits [6]. We consider women that attend one or more ANC visits as an optimistic assumption for those that may receive vaccination in a low-income country setting. Specifically, we assume that 95% of pregnant women attend the first ANC visit with a consistent dropout rate resulting in 46% of women attending four ANC visits. We then adjust this value by assuming only 74% of the women that attend ANC visits will receive vaccination based on the average coverage of other ANC services in Malawi DHS. This is based on the average coverage of ANC services: weighed, blood pressure measured, urine tested, blood sample taken, and nutritional information supplied.

In our base scenario, we assume a donated vaccine presented as a single dose, pre-filled syringe as this may be the most likely avenue for maternal influenza immunization in low-income countries in the immediate future. This vaccine presentation is also likely to result in the lowest wastage. We value the donated vaccine at the lowest price (US$2.90) reported by a low- or lower-middle income country for this vaccine presentation [7]. Due to the single dose presentation, we assume a 5% wastage rate and a 10% buffer stock. The single dose presentation implies the vaccine will require a larger vaccine volume than multi-dose presentations, a value we determined from online sources [8].

We estimated additional cold chain costs for this vaccine by assuming that central level storage would require additional cold rooms; intermediate level storage needs would be met through an even division of cold rooms and refrigerators and health facility cold chain needs would be filled through additional refrigerators. Cold chain volume requirements are based on vaccine volume and the number of expected deliveries at each level per year. The cost per liter of refrigerator space is calculated using the distribution of cold chain equipment from Malawi’s 2011 Cold Chain Assessment which assessed current and future capacity and requirements for new vaccine introductions [9]. The prices per liter for this equipment were taken from the manufacturer [9-18]. Cold room costs per liter were based on PATH’s prior analysis of average cold room costs for WHO Performance, Quality and Safety prequalified equipment [15]. Cold chain equipment is assumed to have a useful life of 10 years but that exceeds the five-year time horizon of the Program Costing Tool. As such, only half of these cold chain costs are attributed during tool’s time horizon.

We assume that the addition of a maternal influenza vaccine will not require additional staff, so service delivery costs are economic costs based on existing staff salaries and three incremental minutes per vaccine. Coverage would be achieved predominantly through ANC clinic visits, though some vaccination would occur during outreach. Our interviews suggested that outreach visits do not occur approximately one-third of the time they are scheduled due to transportation limitations. As such, we include the costs of outreach transportation and the associated personnel per diems for one-third of outreach visits that occur during influenza vaccination season.

Microplanning activities occur in the first year of the program and consist of 4 national meetings, 5 regional meetings and 29 district level meetings. Training would also occur in the first year of the program and consist of one curriculum development workshop, three national level workshops to train the trainers followed by 29 district level training workshops and an additional training at each of the 813 facilities. Subsequent training is assumed to be included in any ongoing EPI/ANC training and results in no incremental program cost. Information, education, and communication (IEC) and social mobilization costs were modelled on the efforts of prior new (infant) vaccine introductions. While the target population is different, this was deemed to be a realistic model as women are already receiving ANC services. Social mobilization consists of four national level events, five zonal events and an additional event in each district and two events at each facility. These events occur in the initial year, though national level communications efforts, and are repeated every other year. Supervision costs include national level visits, district visits as well as monitoring books for the facilities and a post-introduction evaluation. Ten percent of supervision visit costs are allocated to maternal influenza immunization. No additional costs for waste management or disease surveillance are included.

**Table A. Financial and economic costs of maternal influenza immunization program, base with lower ANC coverage.**

| **Activity** | | **Financial Costs 2018-2022, USD (% of total)** | **Economic Costs 2018-2022, USD (% of total)** |  |
| --- | --- | --- | --- | --- |
| Introduction | | 606,813 (61) | 1,191,549 (19) |  |
|  | Microplanning | 159,811 (16) | 304,011 (5) |  |
|  | Training | 150,790 (15) | 460,705 (7) |  |
|  | Social Mobilization/IEC | 76,938 (8) | 176,247 (3) |  |
|  | Cold Chain Supplementation | 219,274 (22) | 250,586 (4) |  |
| Recurrent | | 376,720 (40) | 5,084,834 (82) |  |
|  | Continuing IEC | 55,192 (6) | 55,192 (1) |  |
|  | Service Delivery | 184,225 (19) | 235,172 (4) |  |
|  | Vaccines and Supplies | 6,837 (1) | 4,649,796 (74) |  |
|  | Supervision Monitoring Evaluation | 112,691 (12) | 126,687 (2) |  |
|  | Other Recurrent Costs | 17,775 (2) | 17,987 (1) |  |
| Total Costs | | 983,535 | 6,276,383 | |

Overall financial costs decrease to $1.0 million, primarily due to approximately $200,000 less in cold chain expense associated with fewer vaccines. Vaccine supplies decrease only slightly due to other commodities, but the large decrease in vaccine costs is only an economic cost due to a donated vaccine in this scenario. Service delivery costs also increase as economic costs, but the change does not have a large effect on the total. There are no dramatic changes in annual program costs, though these data are available in S2 Fig and S3 Fig.

**Bibliography**

1. NIHFW, UNOPS, and NIPI. Vaccine and logistics management: health managers modules for immunization. New Delhi, India: National Institute of Health and Family Welfare. http://www.nihfw.org/pdf/NCHRC-Publications/Module%20-%203.pdf

2. WHO. Vaccine stock management: guidelines on stock records for immunization programme and vaccines store managers. Geneva: Immunization, Vaccines, and Biologicals, World Health Organization; 2006. http://apps.who.int/iris/bitstream/10665/69629/1/WHO_IVB_06.12_eng.pdf

3. Malawi NSO. Population projections Malawi. Zomba, Malawi: National Statistical Office of Malawi; 2008. http://www.nsomalawi.mw/images/stories/data_on_line/demography/census_2008/Main%20Report/ThematicReports/Population%20Projections%20Malawi.pdf

4. World Bank. World development indicators. Washington, DC, USA: International Bank for Reconstruction and Development/The World Bank; 2015. http://data.worldbank.org/products/wdi

5. Hirve S, Newman LP, Paget J, Azziz-Baumgartner E, Fitzner J, Bhat N, et al. Influenza sasonality in the tropics and subtropics - when to vaccinate? PLoS One. 2016;11: e0153003.

6. National Statistical Office. Malawi demographic and health survey 2010: final report. Calverton, MD, USA: ICF Macro; 2011. http://apps.who.int/iris/bitstream/10665/250087/1/WHO-IVB-16.07-eng.pdf?ua=1

7. WHO. Vaccine product, price and procurement (V3P) database. Geneva, Switzerland: World Health Organization; 2015. http://apps.who.int/immunization/vaccineprice/en/Navigation/Load?menu=1100

8. World Bank and GAVI Alliance. Immunization financing toolkit; a resource for policy-makers and program managers. Washington, DC, USA: World Bank; 2010. http://siteresources.worldbank.org/HEALTHNUTRITIONANDPOPULATION/Resources/281627-1292531888900/IMMUNIZATIONFINANCINGTOOLKITFINAL121410.pdf

9. Malawi Ministry of Health. Cold chain assessment: inventory of cold chain equipment and assessment of capacity requirements from 2011-2015.
Lilongwe, Malawi: Government of Malawi; 2011.

10. Technology Exchange Lab, Inc. Cambridge, MA, USA: Cambridge Innovation Center; 2016. http://www.techxlab.org/

11. Icelined refrigerators. Esbjerg, Denmark: Vestfrost Solutions; 2016. http://www.vestfrostsolutions.com/icelined-refrigerators/

12. Performance quality safety (PQS) catalogue: prequalified devices and equipment - E003 refrigerators and freezers for storing vaccines and freezing waterpacks (E003/012 Icelined refrigerator, Vestfrost Solutions, MK 404). Geneva, Switzerland: World Health Organization; 2010. http://www.who.int/immunization_standards/vaccine_quality/pqs_e003_012_vestfrost_mk404.pdf

13. ICRC. Emergency medical items catalogue: refrigerator 170L (55L vaccines)/freezer, Sibir V170. Geneva, Switzerland: International Federation of Red Cross and Red Crescent Societies; 2011. http://procurement.ifrc.org/catalogue/detail.aspx?volume=2&groupcode=204&familycode=204001&categorycode=FRIF&productcode=XCOLFRIF05

14. Solar refrigerators. Esbjerg, Denmark: Vestfrost Solutions; 2016. http://www.vestfrostsolutions.com/solar-refrigerators/

15. PATH. Vaccine regional distribution center cost assessment. Seattle, WA, USA: PATH; 2011. http://www.path.org/publications/files/TS_opt_rdc_rpt.pdf

16. WHO. Effective vaccine management (EVM) initiative. Geneva, Switzerland: World Health Organization; 2016. http://www.who.int/immunization/programmes_systems/supply_chain/evm/en/index3.html

17. WHO. Influenza vaccine (seasonal) - inactivated (10 dose vial). Geneva, Switzerland: World Health Organization; 2015. http://www.who.int/immunization_standards/vaccine_quality/pq_239_influenza_seasonal_10dose_sanofi_pasteur/en/

18. Malawi Expanded Programme on Immunisation. Malawi comprehensive EPI multi-year plan 2012-2016.
Lilongwe, Malawi: Government of Malawi; 2011.
